# Effect of Weeping Teats on Intramammary Infection and Somatic Cell Score in Dairy Goats

**DOI:** 10.3389/fvets.2021.622063

**Published:** 2021-07-19

**Authors:** Alessandra Gazzola, Giulietta Minozzi, Stefano Biffani, Silvana Mattiello, Giovanni Bailo, Renata Piccinini

**Affiliations:** ^1^Department of Veterinary Medicine, University of Milano, Milan, Italy; ^2^Consiglio Nazionale delle Ricerche, Istituto di Biologia e Biotecnologia Agraria, Milan, Italy; ^3^Department of Agricultural and Environmental Sciences, Production, Landscape, Agroenergy, Università degli Studi di Milano, Milan, Italy

**Keywords:** goat, weeping teat, udder defect, milk, microbiology, somatic cell count

## Abstract

Mastitis is the most common disease affecting dairy goats and causing economic losses. Although it is accepted that increased somatic cell count (SCC) is mainly a response to infection, its reliability for subclinical mastitis detection in goats is controversial. Indeed, many physiological and extrinsic variables can increase SCC, including breed, parity, age, stage of lactation, seasonal variations, and milking methods. In some animals, milk-secreting tissue is present in the wall of the teat and, in some instances, milk can filter through pores in the skin to the udder surface. This condition is known as “weeping teat” (WT). In these animals, mammary tissue might be prone to develop bacterial infections, although limited information is provided. Weeping teat seems to have a genetic background and is reported to be especially found in goat breeds selected for high milk production. Moreover, it is observed a genetic correlation between WT and decreased milk yield as well as increased somatic cell scores (SCS). Since information on this topic is very limited, this study aimed at investigating any possible relationship between WT, high SCC, and the presence of bacteria in goat milk. Alpine goat farms in Northern Italy were selected based on the presence of WT. Each herd was divided into two age-matched groups, identified as case (WT+) and control (WT–). Half-udder milk samples were collected aseptically at three timepoints; bacteriological analysis was performed, and SCC were determined and transformed in SCS. There was a positive association between SCS and the presence of bacteria in milk (*P* = 0.037) overall, whereas WT udder defect was associated with positive bacterial culture in just one herd (*P* = 0.053). Thus, this herd was further investigated, repeating the sampling and the analysis on the following year. The positive association between high SCS and the presence of bacteria in milk was then confirmed (*P* = 0.007), whereas no association with WT condition was found. These results indicate that WT defect is usually unrelated to both the outcome of milk bacterial culture and SCS. As a side outcome, we could confirm the role of bacterial infection in increasing SCS.

## Introduction

Mastitis is the most common disease affecting dairy goats and represents the main cause of economic losses due to various factors, including the decrease in milk yield and quality as well as the increase of the associated treatment costs.

Somatic cell count (SCC) is the most used indicator of udder health status in cows, but its reliability for subclinical mastitis detection in goats is controversial. Therefore, milk SCC threshold value established for cows is not suitable for goats (1).

Koop et al. (2) showed that high SCC in goat milk is not always associated with a positive bacterial culture. However, various studies reported that increased SCC in goats is mainly a response to infection, thus prevention of IMI can contribute to control SCC in milk (3–5). Rupp et al. (6) provided further evidence that SCC is related to subclinical mastitis in goats, as they observed a positive association between somatic cell score (SCS) and bacterial counts in milk. Moreover, goats with repeated bacteriologically negative udders had the lowest SCS. The degree of the inflammatory reaction may also depend on the microorganisms involved. Rupp et al. (7) detected significant differences between udder halves infected by major pathogens (such as *Staphylococcus aureus, Enterobacteriaceae, Streptococcus* spp., and *Mycoplasma* spp.), and those infected by minor pathogens [such as non-aureus staphylococci (NAS), *Corynebacterium* spp., and *Micrococcus* spp.], which presented lower SCC. In addition, caprine arthritis-encephalitis virus (CAEV) infection was suggested as a possible cause of increased SCC as well (8, 9).

According to Plummer and Plummer (10), in some animals, milk-secreting tissue may be present in the wall of the teat and, in some instances, milk can filter through tiny pores in the skin to the surface of the udder or the teat, in absence of any invisible orifice. This condition is known as “weeping teat” (WT) and is characterized by the presence of milk cysts in goats, in which the accumulated milk may come out to the outside (11). Weeping teat animals can be easily identified by the presence of milk on the outer surface of the udder, especially right before milking. Seykora and McDaniel (12), hypothesized that this condition may contribute to the developing of bacterial infections, as milk, passing through the pores of the skin onto the external surface of the udder, would facilitate the entry of bacteria into the udder itself; therefore, it can be predicted that this porous tissue might be prone to developing bacterial infections and mastitis. Nevertheless, no data on health effects associated to this condition are available. Differently, other two outcomes can occur: milk may communicate with the teat cistern without visual evidence of the presence of this tissue or it may accumulate forming subcutaneous cysts if the secretory tissue does not have an opening.

Currently, very limited data are available on either the frequency of WT or its economic impact in goat farms. In Italy, genetic evaluations for type traits of dairy goats started in 2000, providing information on possible defects with potential functional impact, including the presence/absence of WT. The WT condition is reported to be especially associated with goat breeds selected for high milk production (10, 13). In Italy, mammary gland abnormality has been reported in around 4 and 13% of primiparous Saanen and Alpine females kidding from 2009 to 2014, respectively (14), with an observed incidence, respectively, of 3.6 and 7.5% for primiparous Saanen and Alpine goats. However, this proportion could be underestimated because of voluntary culling or inaccurate evaluation of WT. Biffani et al. (14) observed genetic correlation between WT and milk production or SCS, but the standard error of the estimates was very large. In particular, primiparous Alpine goats showed a loss of 0.046 kg/day milk in comparison with normal does, while SCS increased 0.26–0.21 in pluriparous or primiparous WT animals, respectively (13).

Since information on the role played by WT on the occurrence of intramammary infections is almost unknown, the present case-control study aimed at investigating the possible association between WT, the increase of SCC, and the presence of bacteria in milk of Alpine goats reared in Italy.

## Materials and Methods

### Herds and Sampling

Four Alpine goat farms located in Lombardy region (Northern Italy) and registered in genealogical herd books of Associazione Nazionale della Pastorizia (ASSONAPA, Rome, Italy) were selected based on the phenotypic presence of WT. Alpine goats were chosen as they have a higher frequency of WT compared to the Saanen breed. Herd size ranged from 39 to 116 lactating goats (mean ± *S.E*.: 65.3 ± 17.3). The prevalence of WT in the four herds was 13.6, 14.1, 7.2, and 9.9%. Goats were housed indoor on permanent straw litter, with occasional access to outdoor pasture, and were milked twice/day. All the goats in their second, third or fourth lactation presenting WTs were included as case groups (WT+); the same number of goats, matched with WT+ for age and parity, was recruited as control group (WT–). We decided to exclude parities higher than the fourth one, as older goats show usually more intramammary infections than younger ones. Three WT-goats were culled during the trial period, and therefore were excluded from final analysis. Our follow-up study was performed in 2018, and then repeated in a single herd (herd A) during the following year, to further investigate it.

Half udder milk samples were collected from goats in their second, third, or fourth lactation at three timepoints, at the beginning, in the middle, and at the end of lactation. Samples were taken before milking with an aseptic procedure, by disinfecting the teat with wipes containing chlorhexidine, discarding a few streams of milk from the teat (foremilk), and collecting 10 ml of milk into sterile tubes. After collection, samples were immediately placed on ice and then transported chilled at +4°C to the laboratory.

### Bacteriological Analysis and Somatic Cell Counting

Bacteriological analysis was performed with standard techniques on the day of sampling. In detail, for each sample, 10 μl of milk was plated onto blood agar supplemented with 5% defibrinated bovine blood using a sterile inoculating loop. Plates were then incubated at 37°C and analyzed after 24 and 48 h. Colonies grown on agar plates were isolated and identified following National Mastitis Council guidelines (15), then confirmed by API system. A sample was defined as polymicrobic when more than two distinct colony types were present. The presence of *Mycoplasma* spp. was not investigated, because contagious agalactia of goats is a notifiable disease and no case was officially reported since years. Somatic cell count was determined as well, using a Somacount^TM^ 150 (Bentley Instruments, Minnesota, USA). Cell counts were expressed as cells/μl.

### Statistical Analyses

A general linear mixed model was used to investigate the effect of WT phenotypes and SCC on the observed microbiology outcome (MO).

The general model (Model 1) was:

$$\begin{array}{l} Model\;1:MO=wt+sampling+parity+SCS+animal\\ \;+\;herd+error \end{array}$$

where MO was the dependent variable considered as a binomial trait (0 = no infection, 1 = at least one infected teat); wt (two classes) is the presence/absence of a WT phenotype; sampling (three classes) is the milk sampling at the beginning, the middle, and the end of lactation; parity (three classes) is the parity class; SCS is the mean of the SCC of the two teats transformed to SCS as

$$\begin{array}{l} SCS=Log_{2}\left(\frac{SCC}{100}\right)+3\; \end{array}$$

according to Shook (16); animal is the random permanent environmental effect; herd (4 classes) is the herd where data were collected.

Model 1 was fitted to complete data. Successively, a data subset (A) was created including only records from herd A. The same model, hereinafter called model 2, was fitted after excluding the herd effect. Lastly, dataset A was additionally subset in two datasets, namely B and C. Dataset B included records collected in year 2018, while dataset C included records collected in 2019. Model 2 was fitted to both dataset B and C.

The general linear mixed model was fitted using the function *glmer* of the package “lme4” implemented in the R environment^1^ for statistical programming. Odds ratio have been calculated as exponential of the results of the respective linear general mix model in R statistical environment. All graphical representations were produced using R^1^.

To further corroborate our results, we performed bootstrap resampling to calculate the 95% Confidence Intervals (C.I.) of the estimates of the effects included in the models (17). All bootstrap analyses were performed with the R libraries boot (18, 19), based on 5,000 bootstrap replicates. Further libraries were used, tidyverse, knitr, tidyr, and broom.

## Results

In 2018, a total of 286 half-udder milk samples were collected from 49 Alpine goats (23 cases and 26 controls). The results of bacteriological analysis and SCC determination are shown in Table 1. Overall, most of isolates were NAS (91.7%), the most prevalent being *Staphylococcus chromogenes* (23.6%), *Staphylococcus caprae* (21.8%), unidentified NAS (*Staphylococcus* sp., 20%), and *Staphylococcus epidermidis* (14.5%). Non-aureus staphylococci species varied from herd to herd. In particular, *S. caprae* was isolated mainly in herd A, whereas *S. epidermidis* just in herd D. *S. chromogenes* was equally distributed in three herds, being absent in herd C. *S. aureus* was isolated only in herd C, in two animals. One of them was infected at all the sampling points (goat n. 26), while the other one (n. 31) was positive for *S. aureus* at two supplemental samplings carried out by the farmer and was then culled.

**Table 1:** Results of bacteriological analysis and SCC determination on half-udder milk samples from 49 Alpine goats (23 cases and 26 controls) collected in 2018 at three timepoints (at the beginning, in the middle and at the end of lactation).

				**First sampling**				**Second sampling**				**Third sampling**			
				**Left**		**Right**		**Left**		**Right**		**Left**		**Right**	
**Herd**	**Animal ID**	**No. of lactations**	**Group**	SCC^*^	**Bacteria**	SCC^*^	**Bacteria**	SCC^*^	**Bacteria**	SCC^*^	**Bacteria**	SCC^*^	**Bacteria**	SCC^*^	**Bacteria**
A	1	2	Case	14	/	39	/	306	/	157	*S*. sp.	22	*S. chromogenes*	144	Polymicrobic
A	2	2	Case	82	*S. caprae*	469	/	252	*S. caprae*	250	*S. caprae*	73	/	124	/
A	3	2	Case	288	*S. caprae*	240	*S. caprae*	267	*S. caprae*	254	*S. caprae*	31	/	49	*S. haemolyticus*
A	4	2	Case	13	/	14	/	85	/	578	/	178	*S. chromogenes + S. warneri*	361	/
A	5	2	Case	384	*S*. sp.	622	*S. caprae*	751	*S*. sp.	388	Polymicrobic	256	*S. capitis*	261	/
A	6	3	Case	519	*S. caprae*	171	/	149	/	418	/	508	/	578	*S. chromogenes*
A	7	2	Case	776	/	53	Polymicrobic	8,211	/	1,793	/	255	/	670	/
A	8	4	Case	1,223	/	91	/	781	/	73	/	1,301	/	936	/
A	9	2	Control	43	/	52	/	791	/	554	/	597	/	182	/
A	10	2	Control	1,166	*S*. sp.	364	*E. faecalis*	289	/	*128*	/	297	/	343	/
A	11	2	Control	33	/	27	/	2,577	/	153	/	57	Polymicrobic	61	/
A	12	2	Control	43	/	11	/	86	/	13	/	21	/	16	*S. chromogenes*
A	13	2	Control	1	/	8	/	11	/	7	/	4	/	1	/
A	14	2	Control	103	/	142	/	270	/	370	/	75	/	19	/
A	15	4	Control	79	/	384	*S. caprae*	1,684	/	3,065	/	408	*S. chromogenes*	295	*S. capitis*
A	16	4	Control	n.d.	n.d.	n.d.	n.d.	181	*S. caprae*	93	Polymicrobic	343	/	368	/
A	17	3	Control	168	/	173	/	1,044	/	1,276	/	472	/	351	/
B	18	4	Case	25	/	191	/	4	/	237	/	1,428	/	2,845	/
B	19	4	Case	164	*S*. sp.	370	/	2,988	/	Blind teat		1,602	/	Blind teat	
B	20	4	Case	71	/	50	/	3,185	/	635	/	4,811	/	1,724	/
B	21	4	Control	31	/	45	/	3,787	/	406	/	4,859	/	2,898	/
B	22	4	Control	175	/	181	*S*. sp.	748	/	367	*S*. sp.	882	/	582	/
B	23	4	Control	2,475	*S. chromogenes*	28	/	795	/	171	/	224	/	354	/
C	24	2	Case	28	/	24	/	47	/	106	/	159	/	2,271	/
C	25	2	Case	648	*S*. sp.	115	/	170	*S. capitis*	1,479	/	733	/	953	/
C	26	2	Case	797	/	3,564	*S. aureus*	714	/	534	*S. aureus*	511	*S. capitis*	1,954	*S. aureus*
C	27	3	Control	8	/	8	/	323	*S. capitis*	1	/	473	*S. capitis*	72	/
C	28	2	Control	116	/	80	/	643	/	43	/	2,405		352	/
C	29	2	Control	380	*S*. sp.	277	*S*. sp.	315	/	272	/	665	*S. capitis*	292	/
C	30	2	Control	59	/	54	/	1	/	1	/	35	*S. warneri*	1,363	/
C	31	3	Control	486	*S*. sp.	365	/	1,718	*S. aureus*	127	/	Culled		Culled	
D	32	4	Case	74	/	159	*S. epidermidis*	813	/	5,244	/	231	/	451	*S. epidermidis*
D	33	4	Case	1	/	1	/	145	/	163	/	567	/	125	/
D	34	2	Case	1	/	283	*S. chromogenes*	170	/	375	/	7	/	4	*S. chromogenes*
D	35	2	Case	1	/	4	/	152	/	122	/	10	/	12	/
D	36	2	Case	40	/	7	/	113	/	111	/	22	/	10	*S. chromogenes*
D	37	3	Case	81	*S. epidermidis*	20	/	280	*S. epidermidis*	167	/	557	*S. epidermidis*	32	*S. epidermidis*
D	38	3	Case	51	/	267	/	551	/	704	/	16	/	27	/
D	39	3	Case	1	/	1	/	11	/	11	/	3	/	6	/
D	40	4	Case	192	/	237	/	1,781	/	967	/	614	/	573	*S. chromogenes*
D	41	3	Control	5	/	3	/	82	/	73	/	136	/	186	/
D	42	3	Control	166	/	152	/	19	/	15	/	11	/	8	/
D	43	4	Control	1	/	1	/	38	/	27	/	583	/	134	/
D	44	2	Control	2	/	1	/	1,723	/	414	*S. epidermidis*	433	*S. capitis*	65	*S. chromogenes*
D	45	4	Control	1	/	2	/	118	/	151	/	606	/	752	/
D	46	4	Control	116	*S. caprae*	259	/	51	/	66	/	221	/	308	/
D	47	2	Control	185	/	66	/	41	/	46	/	272	*S. epidermidis*	98	/
D	48	2	Control	95	/	101	/	104	/	197	/	Dried		Dried	
D	49	3	Control	1,010	*S. chromogenes*	239	/	995	/	212	/	4,009	*S. chromogenes*	249	/

* *Expressed as cells/μl; n.d., no data available; S. sp., Staphylococcus specie; /, bacteriologically negative*.

**Figure 1:**
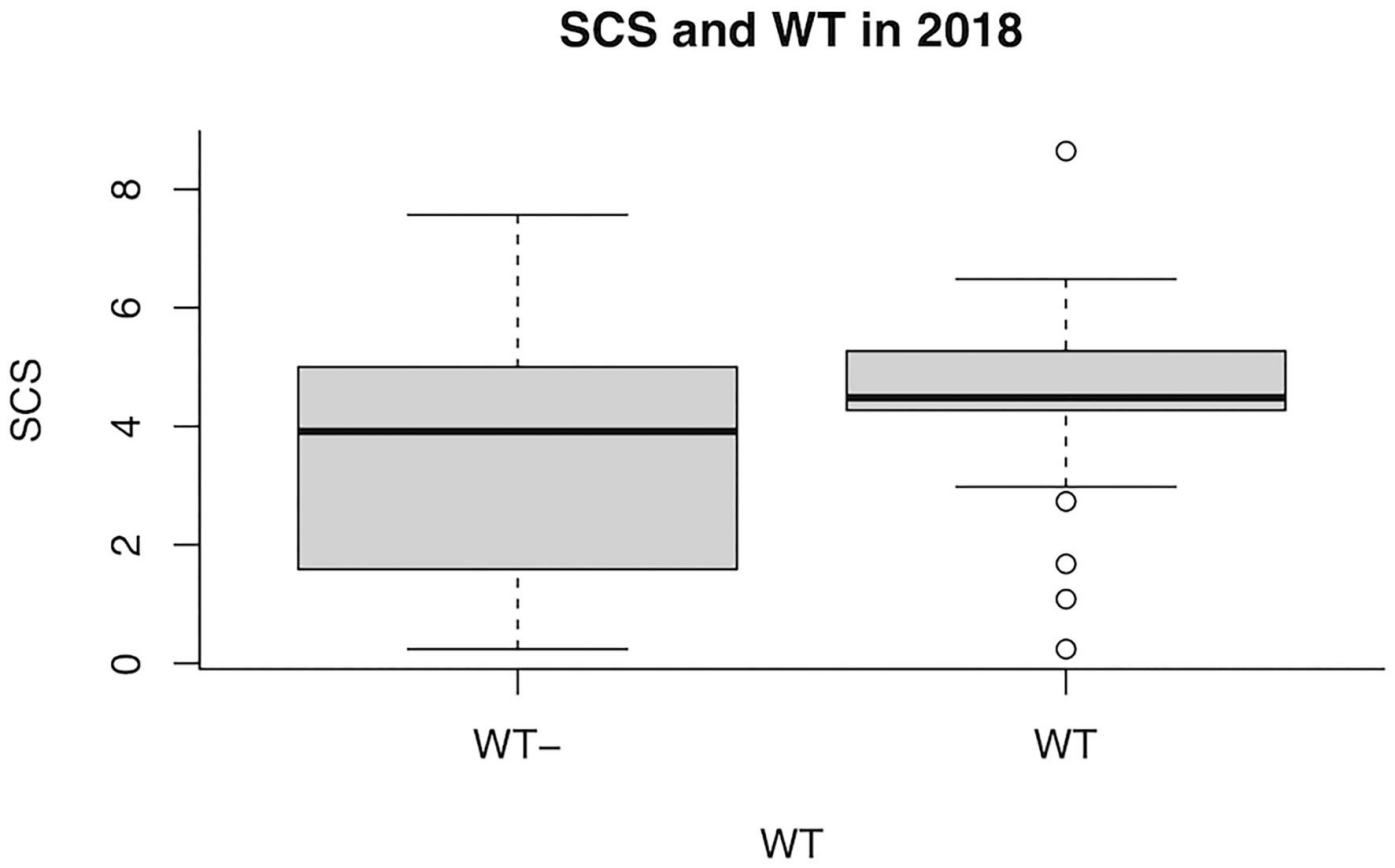
Box plot of somatic cell score (SCS) in goats with (WT+) and without (WT–) weeping teats in herd A in 2018.

Overall SCS mean value in 2018 was 3.8 ± 2.12. Regarding the presence/absence of WT, the SCS-value was 4.1 or 3.5, respectively. When the udder half and the microbiological outcome was considered, the mean value of SCS was 4.6 or 4.3 in the bacteriologically positive left or right half udders, respectively, decreasing to 3.6 or 3.2 in the negative halves. The presence of bacteria in milk was significantly associated with SCS (*P* = 0.037), but neither with WT udder defect, nor to parity or sampling time (Table 2). Considering herd A only, an almost significant effect of WT udder defect on the response to bacterial culture was observed, with a mean SCS-value of 4.4 ± 1.7 in WT udders (median value 4.5) and 3.5 ± 2.3 in normal glands (median value 3.9; *P* = 0.053; Figure 1, Table 3). The box plot of SCS in cases and controls in herd A is shown in Figure 1.

**Table 2:** Results of the linear general mix model on four herds sampled in 2018 on fixed effects in Model 1.

**Effect**	**Odds ratio**	**C.I. LL**	**C.I. UL**	***P*-value**
Weeping teat	2.809	0.934	8.439	0.066
No. of sampling	0.789	0.459	1.357	0.392
No. of lactation	1.068	0.526	2.170	0.853
Somatic cell score	1.284	1.014	1.624	0.037^*^

*Odds ratio, 95% lower confidence intervals (C.I. LL), 95% upper confidence intervals (C.I. UL), and p-values are reported*.

* *P ≤ 0.05*.

**Table 3:** Results of the linear general mix model in herd A sampled in 2018 on fixed effects in Model 2.

**Effect**	**Odds ratio**	**C.I. LL**	**C.I. UL**	***P*-value**
Weeping teat	9.765	0.970	98.313	0.053
No. of sampling	1.223	0.488	3.963	0.660
No. of lactation	0.889	0.199	3.962	0.870
Somatic cell score	1.099	0.669	1.805	0.711

*Odds ratio, 95% lower confidence intervals (C.I. LL), 95% upper confidence intervals (C.I. UL), and p-values are reported*.

Therefore, herd A was further investigated on the following year (2019). A total of 109 half-udder milk samples were collected from 19 goats (9 cases and 10 controls), including the surviving goats sampled in 2018, plus three new animals. The results are shown in Table 4.

**Table 4:** Results of bacteriological analysis and SCC determination on half-udder milk samples from 19 Alpine goats (9 cases and 10 controls) collected in herd A in 2019 at three timepoints (at the beginning, in the middle, and at the end of lactation).

				**First sampling**				**Second sampling**				**Third sampling**			
				**Left**		**Right**		**Left**		**Right**		**Left**		**Right**	
**Herd**	**Animal ID**	**N. of lactations**	**Group**	SCC^*^	**Bacteria**	SCC^*^	**Bacteria**	SCC^*^	**Bacteria**	SCC^*^	**Bacteria**	SCC^*^	**Bacteria**	SCC^*^	**Bacteria**
A	1	3	Case	30	/	49	/	32	/	393	/	197	/	123	/
A	2	3	Case	5,089	/	1,402	/	575	Polymicrobic	720	Polymicrobic	674	*S. caprae*	477	*S. caprae*
A	3	3	Case	163	*S. caprae*	197	*S. caprae*	1,171	*S. caprae*	1,272	*S. caprae*	563	/	507	/
A	4	3	Case	5	/	1,314	*Str*. sp.	n.d.	n.d.	n.d.	n.d.	n.d.	n.d.	n.d.	n.d.
A	5	3	Case	19	/	5	/	428	/	949	/	608	/	631	/
A	6	4	Case	225	/	309	Polymicrobic	468	/	624	*S. epidermidis*	186	/	287	/
A	7	3	Case	283	/	63	/	593	/	820	/	202	Polymicrobic	309	/
A	8	5	Case	4,123	/	84	/	353	/	156	/	215	/	542	/
A	New1	6	Case	39	*S. caprae*	293	*S*. sp.	194	/	546	/	640	/	1,042	/
A	9	3	Control	42	/	28	/	273	/	522	/	136	/	155	/
A	10	3	Control	209	*S. caprae*	blind teat		402	*S. caprae*	373	/	878	*S. caprae*	135	/
A	12	3	Control	142	/	72	*S. chromogenes*	72	/	98	/	197	/	72	/
A	13	3	Control	2	/	4	/	12	/	24	/	1	/	1	/
A	14	3	Control	36	/	79	/	238	/	343	/	185	/	219	/
A	15	5	Control	326	/	439	*S. caprae*	1,790	/	847	*S. caprae*	841	*S. caprae*	797	/
A	16	5	Control	21	/	35	Polymicrobic	53	/	57	/	1	/	5	/
A	17	4	Control	28	/	26	/	635	/	580	/	45	*S*. sp.	3,050	*S. lentus*
A	New2	4	Control	2,887	/	65	/	1,017	*S. caprae*	1	/	712	*S. caprae*	48	/
A	New3	6	Control	114	/	61	/	303	/	283	/	109	/	206	/

*The sampled goats include the surviving goats sampled in 2018, plus three new animals*.

* *Expressed as cells/μl; n.d., no data available; S. sp., Staphylococcus specie; Str. sp., Streptococcus specie; /, bacteriologically negative sample*.

Most of the isolates were NAS (90.5%), identified almost exclusively as *S. caprae* (78.9%). No contagious microorganism was detected.

**Figure 2:**
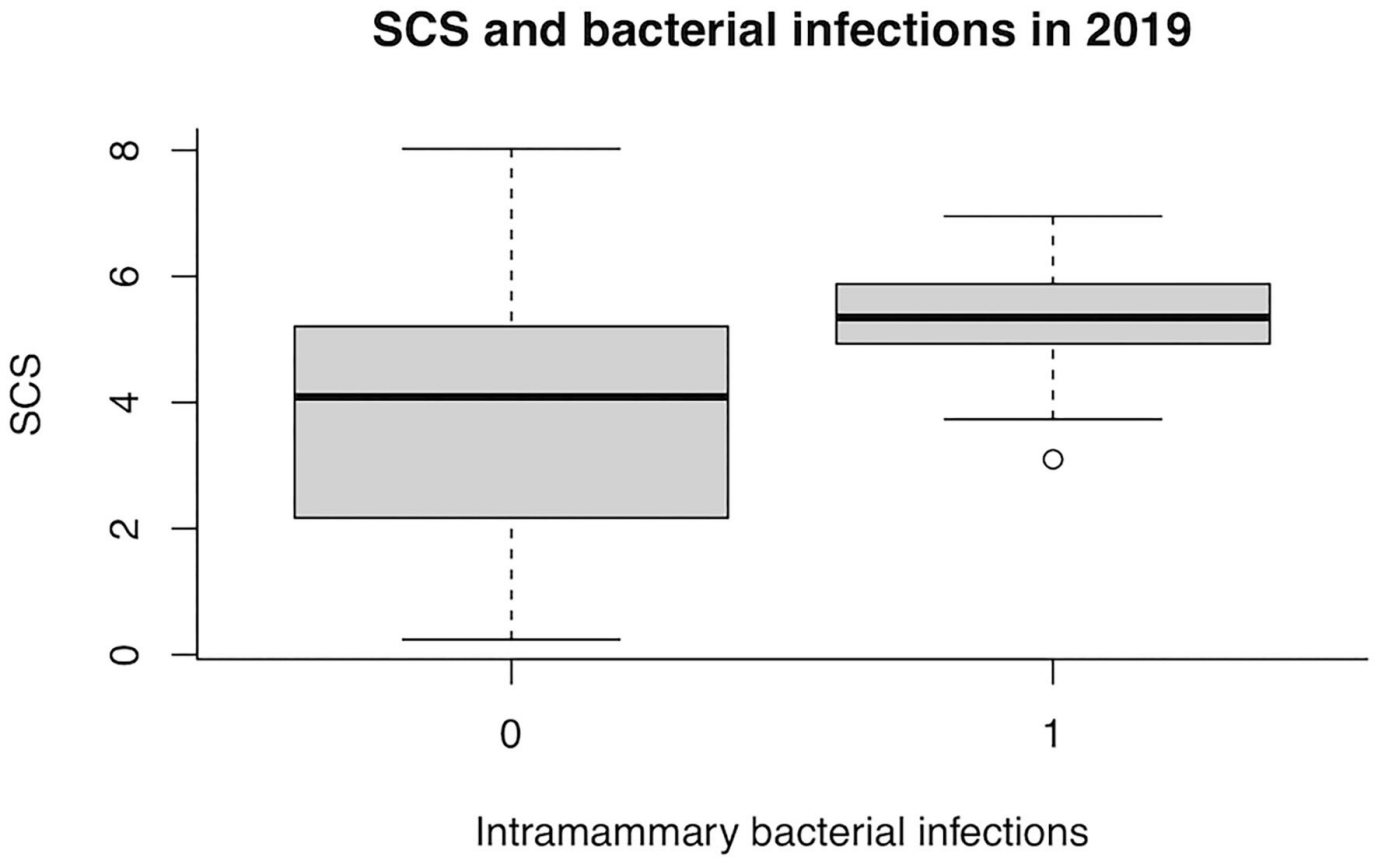
Box plot of somatic cell score (SCS) and bacterial infections in herd A in 2019. 0, no infection; 1, at least one infected teat.

In 2019, the presence of bacteria in milk was no more associated with the WT udder defect, whereas the effect on SCS became statistically significant (*P* = 0.008; Table 5). The box plot of SCS and bacterial infections in herd A in 2019 is shown in Figure 2.

**Table 5:** Results of the linear general mix model on herd A sampled in 2019 on fixed effects in Model 2.

**Effect**	**Odds ratio**	**C.I. LL**	**C.I. UL**	***P*-value**
Weeping teat	0.336	0.081	1.393	0.148
No. of sampling	0.798	0.338	1.886	0.608
No. of lactation	1.161	0.527	2.558	0.709
Somatic cell score	1.847	1.176	2.900	0.008^**^

*Odds ratio, 95% lower confidence intervals (C.I. LL), 95% upper confidence intervals (C.I. UL), and p-values are reported*.

** *P ≤ 0.01*.

Pooling together data collected in 2018 and 2019 in Herd A, no significant effect of SCS nor WT udder on intramammary bacterial infections was observed (Table 6).

**Table 6:** Results of the linear general mix model on herd A sampled in 2018 and 2019 on fixed effects in Model 2.

**Effect**	**Odds ratio**	**C.I. LL**	**C.I. UL**	***P*-value**
Weeping teat	2.198	0.412	11.722	0.366
No. of sampling	1.032	0.554	1.924	0.919
No. of lactation	0.678	0.231	1.991	0.480
Somatic cell score	1.307	0.938	1.821	0.113
Year	1.133	0.127	10.054	0.911

*Odds ratio, 95% lower confidence intervals (C.I. LL), 95% upper confidence intervals (C.I. UL), and p-values are reported*.

The results of the bootstrap C.I. estimates confirmed all previous significant findings. In detail, significant association for SCS levels and MO was confirmed in the analysis of the four Herds sampled in 2018. Furthermore, association between MO and WT was confirmed in herd A in 2018 and between MO and SCS in 2019. All non-significant effects in all models were further confirmed (Supplementary Tables 1–4).

## Discussion

The importance of dairy goats has significantly increased during last decades, providing an alternative to dairy cow products for human consumption (20). A healthy mammary gland is essential for dairy farms and directly correlated with milk yield and quality.

Various pathological conditions of the udder in goats have been described, including supernumerary and abnormal teats, gynecomastia, precocious udders, and fibrocystic disease (11, 21). Among udder defects, information about the WT is extremely limited, but raises concern about its possible role in the development of mastitis. Yet, both its etiology and consequences are not fully understood. In this respect, our study provides new evidence suggesting that WT may be usually unrelated to both the outcome of milk bacterial culture and SCS. However, in one herd out of four we found a positive association of WT defect with positive bacterial culture, although this was not further confirmed in the following year.

In order to define the udder health status, SCC is the most used indicator in cows, but its ability to predict subclinical mastitis in goats has been questioned. Indeed, average SCC values in goats are higher than those in cattle and sheep, since they are influenced by several physiological and environmental factors, such as parity, stage of lactation, season, and milk yield (22–24). Our results highlighted significantly higher SCS in goat udders presenting bacterial infections, independently of parity, season, or managerial factors. This result was in accordance with different studies reporting increased SCC in goats in response to infection (2–5).

Various bacteria can be implicated in goat subclinical mastitis. The most frequently isolated bacteria in the four herds were NAS, mostly *S. chromogenes* and *S. caprae*, followed by unidentified Staphylococci and *S. epidermidis*. Accordingly, in the literature NAS are the most prevalent bacteria isolated from udder halves in goats and appear to behave as minor and opportunistic pathogens (2, 4, 25). Also Koop et al. (2) reported that NAS species have a high prevalence in goat mastitis, and cause persistent infections. Among NAS, *S. caprae* was one of the most frequently isolated Staphylococci, followed by *S. epidermidis, Staphylococcus xylosus, S. chromogenes*, and *Staphylococcus simulans*. Analogously, Rupp et al. (6), reported NAS as the prevalent agents of goat mastitis with a decreasing frequency of isolation from *S. xylosus*, to *S. caprae* and *S. epidermidis*.

## Conclusions

In conclusion, our results cannot confirm the hypothesis that WT udder condition facilitates the entry of bacteria into the udder and that WT goats are more likely to develop localized bacterial infections. However, we cannot exclude that the WT defect could represent a risk for the health of the udder of dairy goats, when associated with particular conditions. Indeed, in our follow-up study only a single herd showed a significant effect of WT on intramammary infection just in the first year, that could not be confirmed in the following year. Additionally, our results showed that the presence of bacteria in milk is positively related with the increase in SCS, despite the physiological increase during lactation. It is necessary to extend the research to a larger number of farms in order to investigate the reasons for this variability and understand if and when the presence of WT could represent a risk for the health of goat's udder.

## Interpretative Summary

Mastitis is the most common disease affecting dairy goats and causing economic losses. Although it is accepted that increased SCSs is mainly a response to infection, its reliability for subclinical mastitis detection in goats is controversial, since it is influenced by many physiological and extrinsic variables, including breed, parity, age, stage of lactation, seasonal variations, and milking methods.

In some animals, milk-secreting tissue is present in the wall of the teat and, in some instances, milk can filter through pores in the skin to the surface of the udder. This condition is known as “weeping teat,” and it is hypothesized that the mammary gland might be prone to develop bacterial infections, although very few information is provided. Our results cannot exclude that the WT defect could represent a risk for udder health of dairy goats, when associated with particular conditions. Indeed, in our follow-up study only a single herd showed a significant effect of WT on intramammary infection, and this was not confirmed by further investigations. As a side outcome, our results showed that the presence of bacteria in milk is positively related with the increase in SCS, despite the physiological increase during lactation.

## Data Availability Statement

The raw data supporting the conclusions of this article will be made available by the authors, without undue reservation.

## Ethics Statement

Ethical review and approval was not required for the animal study because Our study could not be classified as a clinical one and we did not apply interventions outside of routine care, since we just collected milk during routine milking.

## Author Contributions

GM, SB, and RP: design of study and experiments. AG, GB, and SM: laboratory and field activities. AG, GM, SM, and RP: analysis of results, data interpretation, and manuscript drafting. All authors have read and approved the final manuscript.

## Conflict of Interest

The authors declare that the research was conducted in the absence of any commercial or financial relationships that could be construed as a potential conflict of interest.

## Footnotes

^1^www.r-project.org

## References

[1] Persson Y Olofsson I. Direct and indirect measurement of somatic cell count as indicator of intramammary infection in dairy goats. Acta Vet Scand. (2011). 53:15. 10.1186/1751-0147-53-15 21375744 PMC3059284

[2] Koop G De Vliegher S De Visscher A Supré K Haesebrouck F Nielen M. Differences between coagulase-negative Staphylococcus species in persistence and in effect on somatic cell count and milk yield in dairy goats. J Dairy Sci. (2012) 95:5075–84. 10.3168/jds.2012-5615 22916911

[3] Poutrel B de Crémoux R Ducelliez M Verneau D. Control of intramammary infections in goats: impact on somatic cell counts. J Anim Sci. (1997) 75:566–70. 10.2527/1997.752566x 9051481

[4] Bergonier D de Crémoux R Rupp R Lagriffoul G Berthelot X. Mastitis of dairy small ruminants. Vet Res. (2003) 34:689–716. 10.1051/vetres:2003030 14556701

[5] Moroni P Pisoni G Ruffo G Boettcher PJ. Risk factors for intramammary infections and relationship with somatic-cell counts in Italian dairy goats. Prev Vet Med. (2005) 69:163–73. 10.1016/j.prevetmed.2004.10.013 15907567

[6] Rupp R Huau C Caillat H Fassier T Bouvier F Pampouille E. Divergent selection on milk somatic cell count in goats improves udder health and milk quality with no effect on nematode resistance. J Dairy Sci. (2019) 102:5242–53. 10.3168/jds.2018-15664 30904305

[7] Rupp R Clément V Piacere A Robert-Granié C Manfredi E. Genetic parameters for milk somatic cell score and relationship with production and udder type traits in dairy Alpine and Saanen primiparous goats. J Dairy Sci. (2011) 94:3629–34. 10.3168/jds.2010-3694 21700052

[8] Nord K Adnøy T. Effects of infection by caprine arthritis-encephalitis virus on milk production of goats. J Dairy Sci. (1997) 80:2391–7. 10.3168/jds.S0022-0302(97)76190-3 9361211

[9] Sánchez A Contreras A Corrales JJ Marco JC. Relationships between infection with caprine arthritis encephalitis virus, intramammary bacterial infection and somatic cell counts in dairy goats. Vet Rec. (2001) 148:711–4. 10.1136/vr.148.23.711 11430681

[10] Plummer PJ Plummer C. Diseases of the mammary gland. In: Pugh DG, Baird N, editors. Sheep and Goat Medicine. Saunders: Elsevier (2012). pp. 442–465.

[11] Matthews J. Diseases of the mammary gland. In: Diseases of the goat. 4th Ed. Chichester: John Wiley and Sons, Ltd (2016). pp. 198–201.

[12] Seykora AJ McDaniel BT. Udder and teat morphology related to mastitis resistance: a review. J Dairy Sci. (1985) 68:2087–93. 10.3168/jds.S0022-0302(85)81072-9 4044973

[13] Biffani S Minozzi G Piccinini R Castiglioni B Grande S Fresi P. Effect of weeping udder on the level of somatic cell counts and production traits in Italian Alpine and Saanen dairy goats. In: International Bovine Mastitis Conference In Proc. National Mastitis Council. Milan (2018). 10.13140/RG.2.2.19023.38569

[14] Biffani S Tiezzi F Fresi P Stella A Minozzi G. Genetic parameters of weeping teats in Italian Saanen and Alpine dairy goats and their relationship with milk production and somatic cell score. J Dairy Sci. (2020) 103:9167–76. 10.3168/jds.2020-18175 32713699

[15] Hogan JS Gonzales RN Harmon RJ Nickerson SC Oliver SP Pankey JW. Laboratory Handjournal on Bovine Mastitis, revised ed. Madison WI: National Mastitis Council Inc. (1999). P. 222.

[16] Shook GE. Genetic improvement of mastitis through selection on somatic cell count. Vet Clin North Am Food Anim Pract. (1993) 9:563–81. 10.1016/s0749-0720(15)30622-8 8242460

[17] Efron B Tibshirani R. An Introduction to the Bootstrap. New York, NY: Chapman and Hall, Inc. (1993).

[18] Canty A Ripley BD. Boot: Bootstrap R (S-Plus) Functions. R package version 1.3–25. (2020). Available online at: http://CRAN.R-project.org/package=boot (accessed December 27, 2020).

[19] Davison AC Hinkley DV. Bootstrap Methods and Their Applications. Cambridge: Cambridge University Press (1997). Available online at: http://statwww.epfl.ch/davison/BMA/ (accessed January 11, 2021).

[20] Lérias JR Hernández-Castellano LE Suárez-Trujillo A Castro N Pourlis A Almeida AM. The mammary gland in small ruminants: major morphological and functional events underlying milk production–a review. J Dairy Res. (2014) 81:304–18. 10.1017/S0022029914000235 24901899

[21] Yeruham I Sharir B Friedman S Perl S. Cystic dilation of the teat sinuses in doe goats. Vet Rec. (2005) 156:844. 10.1136/vr.156.26.844 15980140

[22] Paape MJ Poutrel B Contreras A Marco JC Capuco AV. Milk somatic cells and lactation in small ruminants. J Dairy Sci. (2001). 84(Suppl.):E237–44. 10.3168/jds.S0022-0302(01)70223-8

[23] Jimenez-Granado R Sanchez-Rodriguez M Arce C Rodriguez-Estevez V. Factors affecting somatic cell count in dairy goats: a review. Span J Agric Res. (2014) 12:133–50. 10.5424/sjar/2014121-3803

[24] Tedde V Bronzo V Puggioni GMG Pollera C Casula A Curone G. Milk cathelicidin and somatic cell counts in dairy goats along the course of lactation. J Dairy Res. (2019) 86:217–21. 10.1017/S0022029919000335 31156071

[25] Dore S Liciardi M Amatiste S Bergagna S Bolzoni G Caligiuri V. Survey on small ruminant bacterial mastitis in Italy, 2013–2014. Small Rumin Res. (2016) 141:91–3. 10.1016/j.smallrumres.2016.07.010

