# Supplementary material for: Effect of Weeping Teats on Intramammary Infection and Somatic Cell Score in Dairy Goats
Source: Front Vet Sci. 2021 Jul 19;8:622063. doi: 10.3389/fvets.2021.622063 (PMC8326401; doi:10.3389/fvets.2021.622063)
Supplement: Supplementary file 1 [file Table_1.DOCX]

**Supplementary Material.**

**Table S.1** Results of 95% Confidence intervals estimated by bootstrap methods on all the 4 Herds in 2018. The table shows 95% lower confidence intervals (C.I.95L) and 95% upper confidence intervals (C.I.95U). Significant effects are highlighted with the “*” symbol.

| Effect (All 4 herds in 2108) | C.I.95L | C.I.95U |
| --- | --- | --- |
| Weeping teat | -0.020 | 2.272 |
| N° of Sampling | -0.834 | 0.306 |
| N° of lactation | -0.672 | 0.841 |
| Somatic Cell Score * | 0.027 | 0.532 |

**Table S.2** Results of 95% Confidence intervals estimated by bootstrap methods on Herd A in 2018. The table shows 95% lower confidence intervals (C.I.95L) and 95% upper confidence intervals (C.I.95U). Significant effects are highlighted with the “*” symbol.

| Effect (Herd A 2018) | C.I.95L | C.I.95U |
| --- | --- | --- |
| Weeping teat* | 0.323 | 36.815 |
| Somatic Cell Score | -0.575 | 1.026 |
| N° of Lactations | -3.497 | 1.945 |
| N° of Sampling | -0.873 | 1.728 |

**Table S.3** Results of 95% Confidence intervals estimated by bootstrap methods on Herd A in 2019. The table shows 95% lower confidence intervals (C.I.95L) and 95% upper confidence intervals (C.I.95U). Significant effects are highlighted with the “*” symbol.

| Effect (Herd A 2019) | C.I.95L | C.I.95U |
| --- | --- | --- |
| Weeping teat | -3.689 | 0.285 |
| Somatic Cell Score* | 0.265 | 1.799 |
| N° of lactation | -0.926 | 1.229 |
| N° of sampling | -1.552 | 0.837 |

**Table S.4** Results of 95% Confidence intervals estimated by bootstrap methods on Herd A in 2018 and 2019. The table shows 95% lower confidence intervals (C.I.95L) and 95% upper confidence intervals (C.I.95U). Significant effects are highlighted with the “*” symbol.

| Effect (Herd A 2018, 2019) | C.I.95L | C.I.95U |
| --- | --- | --- |
| Weeping teats | -0.942 | 2.873 |
| Somatic Cell Score | -0.061 | 0.729 |
| N° of lactation | -0.994 | 0.192 |
| N° of sampling | -0.664 | 0.748 |
| Year | -2.132 | 2.861 |
